# Transmission dynamics of *vivax* malaria in Korea: effectiveness of anti-malarial chemoprophylaxis

**DOI:** 10.1186/1475-2875-13-S1-P29

**Published:** 2014-09-22

**Authors:** Akira Endo, Hiroshi Nishiura

**Affiliations:** 1 The University of Tokyo, Tokyo, Japan

## Background

*Vivax* malaria with two distinct (short- and long-term) incubation periods has been prevalent in the Republic of Korea since its re-emergence in 1993. Chemoprophylaxis has been conducted among military personnel since 1997. We estimated the time-dependent reproduction number, thereby assessing the protective effect of chemoprophylaxis.

## Materials and methods

A mathematical model has been formulated using a renewal equation, estimating the yearly reproduction number (Ry) from 1993 to 2012 by maximum likelihood estimation method. We also computed Akaike Information Criterion (AIC) to test if there was a detectable change point in the trend in relation to chemoprophylaxis.

## Results

Three-year average of Ry showed gradual decline through 1993-2012 with a temporary increase from 2003 to 2005, having been under the threshold 1 since 1998. AIC has suggested that the chemoprophylaxis has cut down Ry by 34% from what it would be without chemoprophylaxis.

## Conclusions

The epidemic of *vivax* malaria in Korea has been brought under control due mainly to mass-chemoprophylaxis.

